# Allergens and Food Additives, Including Potentially Harmful Ones, Present in Food Products that Are Preferred By Children and Adolescents

**DOI:** 10.34763/devperiodmed.20172102.131138

**Published:** 2017-08-11

**Authors:** Sandra Budrewicz, Marcin Banaszczak, Jakub Piotrowski, Maja Czerwińska, Ewa Stachowska

**Affiliations:** 1 Department of Biochemistry and Human Nutrition, Pomeranian Medical University in Szczecin, Szczecin, Poland

**Keywords:** children’s habits, potentially harmful substances, food, nawyki dzieci, substancje potencjalne szkodliwe, żywność

## Abstract

**Introduction:**

The proper development of a child is linked with proper nutrition, including nutritional habits which are formed from childhood.

**The aim of the study:**

The aim of the study was to establish a list of the most popular food products among children and to develop a register of potentially dangerous substances on a Facebook website.

**Materials and methods:**

A website was created on Facebook. The participants provided lists of favorite dishes or products.

**Results:**

The study involved 264 participants. An inverse correlation was observed with reference to the age of the subjects and the occurrence of sugar syrup in their diet (R=-0.20; p<0.001), glucose-fructose (R= -0.18; p< 0.004), and glucose (R=-0.13; p< 0.039) syrups. The most common potential food allergens are: gluten (R=0.28; p<0.001), eggs (R=0.28; p<0.001), and wheat (R=0.25; p<0.001). The main substances added to food that are present in a child’s diet that increase proportionally with reference to the child’s age are: salicylates (R=0.37; p<0.001), iron and ammonium sulfates (R=0.21; p<0.001).

**Conclusion:**

The choices of favorite products are related to age and sex. Products containing gluten, the consumption of which increases with age, carry a risk of undiagnosed celiac disease and non-celiac gluten sensitivity in people with a genetic predisposition. Facebook has fulfilled its role as an effective tool for gathering information about the food preferences of children and adolescents.

## Streszczenie

**Wstęp:**

Prawidłowy rozwój dziecka związany jest z właściwym sposobem żywienia, w tym nawykami żywieniowymi, które kształtują się we wczesnym dzieciństwie.

**Cel badania:**

Celem badania było stworzenie listy najpopularniejszych wśród dzieci produktów spożywczych oraz opracowanie zbioru potencjalnych alergenów w nich występujących przy użyciu strony internetowej Facebook.

**Materiały i metody:**

Na profilu społecznościowym Facebook utworzono stronę internetową, na której uczestnicy przedstawili listę ulubionych potraw lub produktów spożywczych.

**Wyniki:**

W badaniu wzięło udział 264 uczestników. Zaobserwowano odwrotną korelację pomiędzy wiekiem badanych a częstością występowania w ich dietach cukru (R=-0,20; p<0,001), syropu glukozowo-fruktozowego (R= -0,18; p< 0,004) oraz syropu glukozowego (R=-0,13; p<0,039). Najczęstszymi potencjalnymi alergenami pokarmowymi były: gluten (R=0,28; p<0,001), jaja (R=0,28; p<0,001) oraz pszenica (R=0,25; p<0,001). Odnotowano korelację pomiędzy dodatkami do żywności a wiekiem dziecka. Głównymi dodatkami do żywności zawartymi w diecie dzieci były salicylany (R=0,37; p<0,001), siarczan żelaza i siarczan amonu (R=0,21; p<0,001).

**Wnioski:**

Wybór ulubionych produktów żywnościowych związany był z wiekiem i płcią badanych osób. Produkty zawierające gluten, którego spożycie wzrasta wraz z wiekiem, może nieść za sobą ryzyko ujawnienia się niezdiagnozowanej celiakii oraz nieceliakalnej nadwrażliwości na gluten u osób z predyspozycją genetyczną. Strona internetowa Facebook sprawdziła się jako skuteczne narzędzie do gromadzenia informacji o preferencjach żywieniowych dzieci i młodzieży.

A balanced diet is most important when a child is growing and maturing [1]. During this time, the need for nutrients is the highest [2, 3]. The amount of nutrients and their proportions in a child’s diet is carefully regulated by national nutrition societies. In Poland this role is fulfilled by the Polish National Food and Nutrition Institute. The Institute develops the rules of healthy nutrition for children and teenagers (http://www.izz.waw.pl/en/struktura-instytut)

Farming techniques in relation to both plants and animals as well as the methods of food production involve substances (such as preservatives and dyes) that could be harmful for the organism or that have an allergenic potential [4, 5]. The RAK Foundation for Nutritional Awareness is responsible for the monitoring of such substances. It is a worldwide organization that aims to raise the awareness about nutrition and spreading information about potentially harmful substances. Its database includes over 100 000 food products. Every entry has a description of allergens and harmful or controversial ingredients added to a given food product (www.foodfacts.com)

Children’s eating preferences are developed on the basis of genetic and environmental factors [6]. Children are good at learning and accepting new tastes and food products [7, 8]. During kids’ first years of life parents play a key role in the development of their child’s eating preferences [9]. When children become older, their taste is more and more influenced by their peers and the media [10]. The latter include a large number of advertisements, special TV channels and websites that significantly influence children [11]. Nearly all food companies and fast food restaurants have websites with links to products for parents, children and teenagers [12, 13]. Food companies that are recognized all over the world often support sports events and the most popular sportsmen advertise their products [12, 14]. This phenomenon strengthens the positive image of these companies [15]. It has been proven that with age children get better at recognizing the labels of food companies [11].

The Internet is one of the most important methods of communication for children and teenagers. The fast and unlimited access to information granted by the Internet gives it a significant advantage over traditional media, such as the press, TV or radio. Young people want to be independent in their search for new and interesting information and they want to do it at a time and place convenient for them. Traditional ways of using the Internet (browsing websites in search of information, etc.) are being replaced by social media websites that gather thousands – if not millions – of people with similar interests and expectations and that make it possible to create new information which is shared and used in lively discussions [16, 17].

## The aims of the study

The aims of the study are:

– to create a list of products that are most willingly consumed by children and teenagers;
– to create a list of typical allergens and possibly harmful substances present in the products that are preferred by children.

## Materials and methods

### 1 The group studied

A group called “Babyshambles children Top 10 favorite food choices” was created on Facebook. 263 people took part in the study. They sent data on their favorite meals through the social media website. The participants were between 1-18 years old (tab. I). The study included 158 responses written by girls (or their parents), and 105 responses written by boys (or their parents). The study protocol was approved by the ethics committee of the Pomeranian Medical University and conformed to the ethical guidelines of the 1975 Declaration of Helsinki.

### 2 Data collection and the verification of potentially harmful substances

The participants (or their guardians), who had a Facebook account, sent their responses by electronic means providing the following information: their names, age, and a maximum of 10 of their favorite dishes or food products. A table of potentially harmful dishes and food products was created by means of Microsoft Excel. The analysis of the harmful food additives in the specified products was carried out with the help of the data included in the RAK Foundation for Nutritional Awareness’ database. If a particular dish or product was not described in the database, its content was analyzed on the basis of its label or the typical content of a dish. If the product or dish included potentially harmful substances, they were included in the database.

**Table I:** Study group characteristic. Tabela I. Charakterystyka grupy badanej.

Age *Wiek*	Number of Girls *Liczba dziewcząt*	Number of Boys *Liczba chłopców*
1-3	23	17
4-6	33	32
7-12	19	7
13-16	52	30
17-18	31	19
Total ***Całkowita liczba***	**158**	**105**

### 3 Statistical analysis

The relationships between each variable were analyzed by means of Spearman’s rank correlation coefficient and descriptive statistics. The value p≤0.05 was considered statistically significant. The results were developed by means of Statistica 10 (Statsoft Poland).

## Results

### The top 10 products chosen by children and teenagers regardless of age and gender

The ten most popular dishes and products are: French fries (27%), chocolate (27%), pizza (23%), chicken (21%), bananas (20%), tomato soup (19%), pancakes (17%), spaghetti (16%), apples (16%), and fish (16%) − table II.

The top potentially harmful substances that endanger children’s health regardless of their age and gender

**Fig. 1:**
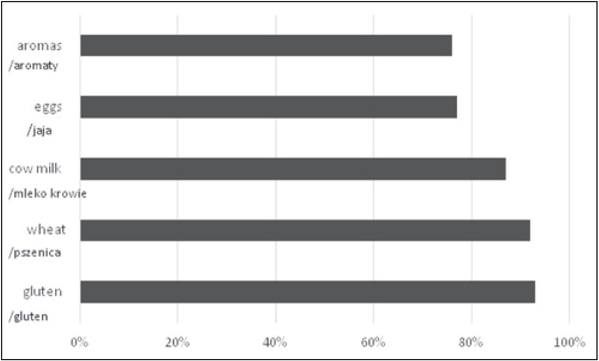
The most popular potential allergens in children’s products.

The potentially harmful substances included in these products are: gluten (93%), wheat (92%), cow’s milk (87%), eggs (77%), and aromas (76%) (fig. 1).

**Table II:** Top 10 the most popular products consumed by children and adolescents. Tabela II. Top 10 najpopularniejszych produktów spożywanych przez badane dzieci i młodzież.

Products *Produkty*	Usage in grup [%] *Częstość w grupie*
French fries *Frytki*	27%
Chocolate *Czekolada*	27%
Pizza *Pizza*	23%
Chicken *Kurczak*	21%
Bananas *Banany*	20%
Tomato soup *Zupa pomidorowa*	19%
Pancakes *Naleśniki*	17%
Spaghetti *Spaghetti*	17%
Apples *Jabłka*	16%
Fish *Ryby*	16%

### Product preference in relation to gender

The results show that girls (82%) more often than boys (70%) chose eggs and products that included eggs (R=-0.13; p<0.037) – tab III. Boys preferred products that were prepared on the basis of palm oil (24% boys vs. 12% girls) (R=0.15; p< 0.017) and products that included sesame (17% vs. 9% respectively) (R=0.13; p<0.042) − table III.

**Table III:** Preference of consumed products and additional substances included in this food, including potentially harmful, depending on the gender of examined persons. Tabela III. Preferencja spożywanych produktów oraz zawartych w tej żywności dodatkowych substancji, w tym potencjalnie szkodliwych, w zależności od płci badanych osób.

Products and food additions *Produkty i dodatki do żywności*	Percent of population Boys *Odsetek populacji chłopców*	Percent of population Girls *Odsetek populacji dziewczynek*	R	P value
Gluten *Gluten*	90%	94%	-0.07	NS
*Wheat* Pszenica	89%	93%	-0.07	NS
Eggs *Jaja*	70%	82%	-0.13	<0.038
Sesame *Sezam*	17%	9%	0.13	<0.042
*Olej* Palm *palmowy* oil	24%	12%	0.15	<0.017
Soy *Soja*	39%	40%	-0.005	NS
Tomatoes *Pomidory*	56%	56%	0.002	NS
*Orzechy* Hazelnuts *laskowe*	33%	34%	-0.015	NS
Mustard plants *Gorczyc*a	55%	60%	-0.039	NS
Cocoa paste *Pasta kakaowa*	40%	44%	-0.039	NS
Chili *Chili*	3%	6%	-0.055	NS
Vanilla aroma *Aromat waniliowy*	37%	38%	-0.015	NS
Sugar syrup *Syrop cukrowy*	10%	8%	0.027	NS
** Glucose syrup *Syrop glukozowy*	17%	11%	0.072	NS
Glucose-fructose syrup Syrop glukozowo-fruktozowy	27%	32%	-0,046	NS
BHT (butylated hydroxytoluene) *Butylohydroksytoluen*	13%	5%	0.13	<0.032
Monosodium glutamate *Glutaminian sodu*	42%	49%	-0.055	NS
Sodium benzoate and potassium sorbate Benzoesan sodu i sorbinian potasu	9%	16%	-0.099	NS
Sulfates *Siarczany*	52%	44%	0.082	NS
Calcium disodium EDTA (E385) *Sól wapniowo-disodowa*	1%	4%	-0.087	NS

### The consumption of potentially harmful substances in relation to gender

The analysis shows that boys (13%) more often than girls (5%) are exposed to products that include butylated hydroxytoluene (BHT) (R=0.13; p<0.032). The study did not provide information on the potentially harmful substance that would be characterized by higher preference in the group of girls (tab. III).

### Preferences in relation to the age of the children

There was a positive correlation between the age of the children and their preference in relation to products and dishes that included gluten (R=0.28; p<0.000), and products and dishes that included wheat (R=0.25; p<0.000) − table IV. A positive correlation in reference to age was also observed for dishes and products that included eggs (R=0.28; p<0.000005), mustard plant (R=0.204; p<0.001), chili (R=0.200; p<0.001), and soy (R=0.171; p<0.005). The largest amount of soy products and dishes is preferred by children aged 7-12 (73%) - table IV. The study revealed a disturbing phenomenon that there was a negative correlation between the age of a child and the consumption of products that included sugar syrup (R=-0.20; p<0.001), glucose-fructose syrup (R=-0.18; p<0.004), and glucose syrup (R=-0.13; p<0.039). There was also a negative correlation in reference to age and the consumption of dishes and products that included cocoa paste (R=-0.16; p<0.008) − table IV.

**Table IV:** Preference of consumed products and additional substances included in this food, including potentially harmful, ones depending on the age of the persons examined. Tabela IV. Preferencja spożywanych produktów oraz zawartych w tej żywności dodatkowych substancji, w tym potencjalnie szkodliwych, w zależności od wieku badanych osób.

Products by age *Zależność między produktem a wiekiem*	R	P value
Gluten *Glute*n	0.28	0.000005
*Wheat* Pszenica	0.25	0.000031
Eggs *Jaja*	0.28	0.000005
Sesame *Sezam*	0.05	NS
*Palm oil* *Olej palmowy*	-0.06	NS
Soy *Soja*	0.17	0.005
*Tomatoes* Pomidory	0.37	0.001
Hazelnuts *Orzechy laskowe*	-0.15	0.014
Mustard plants *Gorczyca*	0.20	0.001
Cocoa paste *Pasta kakowa*	-0.16	0.008
Chili *Chili*	0.20	0.001
** Vanilla aroma *Aromat waniliowy*	-0.16	0.012
Sugar syrup *Syrop cukrowy*	0.20	0.001
Glucose syrup *Syrop glukozow*y	-0.13	0.039
Glucose-fructose syrup *Syrop glukozowo-fruktozowy*	-0.18	0.004
BHT (butylated hydroxytoluene) *Butylohydroksytoluen*	-0.14	0.024
Monosodium glutamate *Glutaminian sodu*	0.19	0.003
Sodium benzoate and potassium sorbat *e Benzoesan sodu i sorbinian potasu*	0.19	0.002
*Sulfates* Siarczany	0.21	0.001
Calcium disodium EDTA (E385) *Sól wapniowo-disodowa*	0.14	0.021
Salicylates *Salicylan*y	0.37	0.001
Iron sulphate and ammonium sulfate *Siarczan żelaza i siarczan amonu*	0.27	0.001

The main potentially dangerous substances and allergens in relation to the age of children

The main potentially dangerous substances in terms of consumption in relation to the age of children are salicylates (R=0.37; p<0.001), iron sulphate and ammonium sulfate (R=0.27; p<0.001), monosodium glutamate (R=0.19; p<0.003), sodium benzoate and potassium sorbate (R=0.19; p<0.002), vanilla aroma (R=-0.16; p<0.012), the allergen of hazelnuts (R=-0.15; p<0.014), BHT (R= -0.14; p<0.024), and calcium disodium EDTA (E385) (R=0.14; p<0.021) – table IV.

## Discussion

The number of people who use social media websites has been growing in recent years. One of the most popular examples of such websites is Facebook. Nowadays it serves as an important method of communication with friends. People share their personal information on Facebook. Taking into account the popularity of this social networking site and the speed of data shared by means of the Internet, it seems that Facebook fulfilled its role in terms of the collection of data on the nutritional preferences of children and teenagers.

The analysis of correlations between gender and potentially harmful substances yielded interesting results. Among boys the results pointed to significant amounts of BHT, sesame, and palm oil. In the boys’ diet, a popular brand of chocolate corn flakes and chewing gum seem to be the sources of BHT, whereas sesame and palm oil were present in such products as halva, cookies, hamburgers, and toast. Girls preferred dairy products (eggs), and any dishes that included eggs: “paszteciki” (special pastries, i.e. deep-fried dough stuffed with meat or vegetarian filling), dumplings, cakes, croquettes, “pierogi” (a Polish type of dumplings), waffles, “knedle” (a different Polish type of dumplings), “łazanki” (a type of pasta dish), “kopytka” (a type of potato dumplings), pancakes, and other starch dishes. The results of this study correspond to those of Japanese scientists who in 2014 carried out an experiment referring to the differences in the nutritional preferences of school-age children in relation to gender. The study showed that boys have a greater preference for fat than girls [18]. The study of 2014 proved that childhood preferences are influenced by genes and the people around the children. The latter strongly develop preferences towards products of high energetic density and those that include starch [19].

The analysis of correlations between the content of the children’s favorite foods and their age also yielded interesting results. Younger children preferred products that included glucose-fructose syrup, glucose syrup, sugar syrup, vanilla aroma, hazelnuts, and cocoa paste. These were usually ready-made products of popular brands which were high in calories and had little nutritional value: homogenized cheese, yoghurts, candy, lollipops, croissants, chocolate, sweet drinks, and chocolate butter. These products are widely advertised on every TV channel. Commercials of highly processed foods often aim directly at children [20]. Frequently the main characters of these commercials are cartoon heroes or popular sportsmen. The child may want to follow the characters’ example and persuade his or her parents to buy the advertised products [21]. In this way, the correlations between the content of allergens and the age of children can be explained. It is also worth mentioning that the participants of this study directly pointed out specific names of food products. After the examination of the labels of these products it turned out that most of them are manufactured by a single, well-known company [11, 15, 20].

Older children preferred dishes and food products that included calcium disodium EDTA (E 385), sodium glutamate, soy, preservatives (sodium benzoate and potassium sorbate), sulphates of ammonium and iron, starch, eggs, salicylates, and chili. Sodium glutamate was present in dishes served at Chinese restaurants. EDTA (E 385) in mayonnaise, which is widely used in many types of fast food. It seems that in this case the positive correlation is not accidental, either. The preference for the consumption of these dishes and food products starts to increase when the child goes to school (starting at the age of 7). Both the environment and the child’s peers may have a significant influence on this phenomenon. At this age children face a new reality and new challenges appear.

The analysis of the data showed that the top 10 favorite dishes and food products were: French fries (27%), chocolate (27%), pizza (23%), chicken (21%), bananas (20%), tomato soup (19%), pancakes (17%), spaghetti (17%), apples (16%), and fish (16%). Fast food products were high on the top 10 list (first place – French fries 27%, third place – pizza 23%. The results correspond to those reached by Polish scientists [21]. In that study fast food products were ranked second on the list of favorite foods. The same study revealed that most children consider frequent visits at popular fast food restaurants very pleasant, because the food is tasty and their parents allow them to choose what they want to eat. In this case we see an element of child independence [21].

One of the most favorite food products consumed by children and teenagers was chocolate (26%). The anticipated high position (second place) of chocolate can be the result of an innate preference for the sweet taste [8]. The younger the age group, the larger the number of products that included the following ingredients: vanilla aroma, glucose-fructose syrup, glucose syrup, and sugar syrup [22]. The early introduction of products that are high in calories and low in nutritional value is a very disturbing phenomenon in the light of the widespread obesity which is now becoming a global epidemic [23].

Among the top ten favorite dishes and food products there was also a place for fruit [24] – in our study bananas were ranked fifth, and apples ninth. In a similar study in Poland - most children (92%) preferred apples most of all the mentioned types of fruit [22]. Others Polish authors also revealed that apples were the most frequently consumed type of fruit [24]. It is also worth mentioning that among the favorite products chosen by the participants there were no raw vegetables (as a separate food product or in the form of salads). 17.5% of the participants prefer tomato soup (sixth place on the list).

As suggested by the EFSA Panel on Dietetic Products, Nutrition, one should eat two portions of products that are a source of protein (meat, fish, eggs) [25]. Descriptive analysis revealed that 16% of the participants liked fish (with salmon being mentioned most frequently). There were no statistically significant differences in relation to the preferences of fish consumption and the age or gender of the participants. Statistical analysis revealed that the preference for the consumption of products and dishes that include soy increases with age. The frequency of the appearance of a general allergy to soy in the population is not known yet. However, it seems that it depends on the exposure to this type of food and on nutritional habits. Soy proteins are widely used in child nutrition products and in highly processed food products. This is why the number of soy allergies is growing [26].

The study revealed that gluten, wheat, cow’s milk, eggs, and aromas are among the most frequently occurring allergens and substances that can be potentially harmful for the organism Fig.1. The results correspond to those reached by Sicherer and Samspon. In that study, cow’s milk, eggs, peanuts, wheat, soy, nuts, fish, and seafood were among the products that most often cause allergies among children. It is pointed out that cereal proteins are an important allergen among children [27]. Statistical analysis did not reveal statistically significant differences in relation to the gender or age of the participants and the preference for the consumption of dishes and food products that include wheat, gluten, cow’s milk, corn, peanuts, mushrooms, thickeners, leavening agents, and monoglycerides and diglycerides of fatty acids. Perhaps if the group of participants had been larger, statistical significance would be achieved and it would show the differences between the preferences for consumption and age or gender.

Facebook proved to be a reliable tool for the collection of information about the nutritional preferences of children and teenagers. Loshe and Wamboldt also used Facebook in order to collect data for their study, and they considered the social media website a more effective and more efficient tool in comparison to traditional methods [28].

In the future, a step forward would be to use Facebook in order to educate parents in terms of rational nutrition and psychoeducation.

## Conclusions

1. The following conclusions were reached on the basis of the research conducted:
2. Early introduction of widely advertised food products that contain glucose-fructose syrup may contribute to the promotion of obesity and metabolic disorders.
3. Another disturbing fact is the rise in the consumption of monosodium glutamate and preservatives, as it points at the consumption of highly processed food that has low nutritional value.
4. The choices of favorite types of food are related to age and gender. School children are highly influenced by their peers when it comes to the choices of favorite dishes.
